# Living alone and the risk of depressive symptoms: a cross-sectional and cohort analysis based on the China Health and Retirement Longitudinal Study

**DOI:** 10.1186/s12888-023-05370-y

**Published:** 2023-11-17

**Authors:** Guangjun Zheng, Biying Zhou, Zhenger Fang, Chunxia Jing, Sui Zhu, Mingliang Liu, Xia Chen, Lei Zuo, Haiyan Chen, Guang Hao

**Affiliations:** 1 Department of Public Health and Preventive Medicine, School of Medicine, Jinan University, Guangzhou, China; 2 Guangdong Key Laboratory of Environmental Exposure and Health, Jinan University, Guangzhou, China; 3 Department of Parasitic Disease and Endemic Disease Control and Prevention, Guangzhou Center for Disease Control and Prevention, Guangzhou, China; 4 Guangdong Provincial Engineering Research Center of Public Health Detection and Assessment, School of Public Health, Guangdong Pharmaceutical University, No. 283 Jianghai Avenue, Guangzhou, 510632 Guangdong China

**Keywords:** Living alone, Depressive symptoms, Older population, Financial support

## Abstract

**Background:**

There were a few studies that examined the longitudinal association between living alone and depressive symptoms, and the vast majority of them were conducted in patients with certain diseases, such as heart failure, cancer, and glaucoma. This study aimed to examine the association between living alone and depressive symptoms in a large representative older Chinese population.

**Methods:**

The China Health and Retirement Longitudinal Study (CHARLS) data from 2015 to 2018 were used. Living alone was defined as participants who did not live with others ever or more than 11 months in the past year at baseline. Depressive symptoms were measured using the 10-item Center for Epidemiological Studies-Depression Scale (CES-D10). The multivariate logistic regression was used to estimate the relationship between living alone and depressive symptoms.

**Results:**

There were 5,311 and 2,696 participants ≥ 60 years old included in the cross-sectional and cohort analysis, respectively. The risk of depressive symptoms in participants who lived alone was significantly higher than those who lived with others in both cross-sectional (OR:1.33; 95%CI:1.14,1.54) and cohort analysis (OR:1.23; 95%CI:0.97,1.55). There was a significant interaction between financial support and living alone (*P*_interaction_ = 0.008) on the risk of depressive symptoms. Stratified analyses showed that, compared to those who lived with others, the risk of depressive symptoms in participants who lived alone increased by 83% (OR:1.83; 95%CI:1.26,2.65) in participants receiving lower financial support. However, we did not find statistically significant associations in participants with medium (OR:1.10; 95%CI: 0.74,1.63) and higher financial support (OR: 0.87; 95%CI: 0.53,1.41).

**Conclusion:**

Living alone was associated with a higher risk of depressive symptoms in the Chinese older population, and this association was moderated by the receipt of financial support. Living alone may be an effective and easy predictor for early identification of high-risk populations of depression in the older population.

**Supplementary Information:**

The online version contains supplementary material available at 10.1186/s12888-023-05370-y.

## Introduction

Depression is a significant global public health issue [1–3], which will become the second main cause of disease burden by 2030 [4]. Depression also is an important risk factor for cardiovascular disease, cancer, and all-cause mortality, especially in the elderly population (also known as later-life depression) [5–7]. It was reported that more than 34 million all-age disability-adjusted life-years were associated with depression [8]. Psychosocial factors, such as bereavement, lack of social support, and some other negative life events are important factors of depression in older people [9–12].

Living alone, a special residential style due to being unmarried or widowed and some other reasons, can increase the risk of social isolation, loneliness, and malnutrition, especially in the older population [13]. Studies have shown that living alone or loneliness is linked to the risk of obesity, cardiovascular diseases, stroke, and premature death [14–17]. Several studies examined the association between living alone and the risk of depressive symptoms [18–24], but the results were controversial [25, 26]. There were a few longitudinal studies that examined the association between living alone and depressive symptoms. A recent meta-analysis including six cohort studies and one case-control study found that those who lived alone faced a 1.42 times higher risk of depressive symptoms compared with those who had other living arrangements [27]. The vast majority of included cohort studies were conducted in patients with certain diseases, including heart failure, cancer, glaucoma, post-myocardial infarction, or mental illness [28–33]. Further, prior studies suggested that specific risk factors (such as sex, socioeconomic status, social support, and living in urban/rural areas) may modify the effect of living alone on depressive symptoms, but the results were controversial [19, 34]. On the other hand, older people may have other diseases (e.g., dementia) that cause difficulties for psychiatrists or geriatricians to prescribe adequate drugs to treat depressive disorder [35, 36]. Living alone may be an effective and easy predictor for early identification of high-risk populations of depression in the older population, which is of utmost importance to delay and prevent depression and reduce the social and economic burden. Therefore, this study mainly aimed to investigate the longitudinal association between living alone and depressive symptoms in the general population 60 years and older and to explore the potential effect modifications.

## Methods

### Study participants

The data was derived from the China Health and Retirement Longitudinal Study (CHARLS), which is a nationally representative longitudinal survey [37]. The CHARLS participants were sampled using a multistage probability sampling strategy and probability proportionate to the size sampling method. It covered 150 counties of 28 provinces, municipal cities, and autonomous regions of China [37]. The baseline survey of the CHARLS was conducted in 2011 and it followed up every two to three years. The CHARLS was approved by the Ethical Review Committee at Peking University, and all participants signed informed consent before participation.

**Fig. 1:**
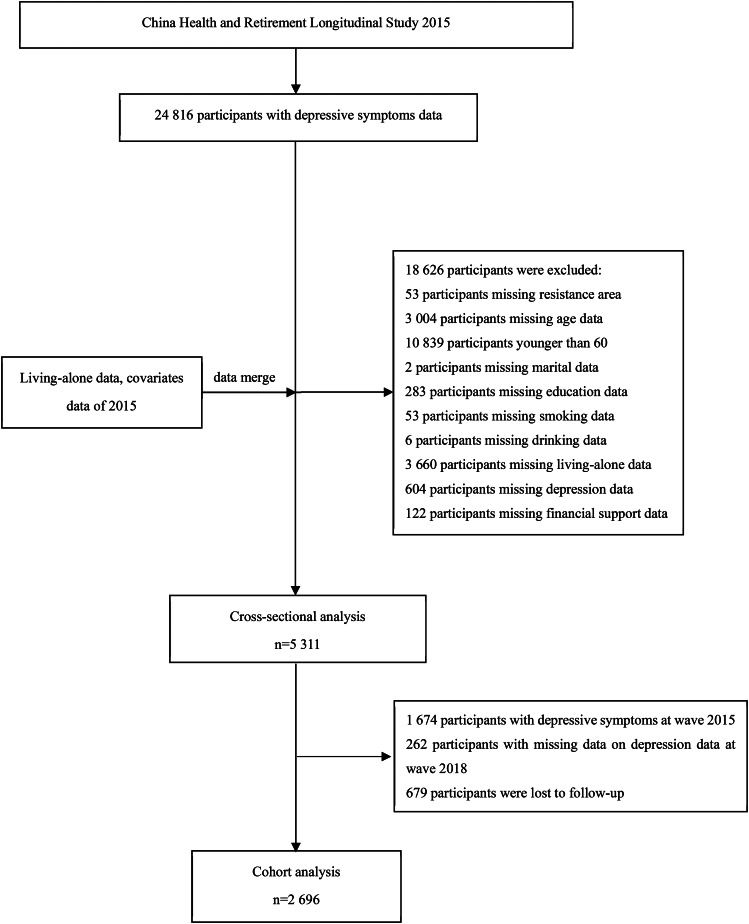
The flow chart of study participants

In this study, the data from waves 2015 and 2018 were used. In the cross-sectional analysis, a total of 5,311 participants with complete data on depressive symptoms, living arrangement, and selected covariates were included. In the longitudinal analysis, we further excluded the 1,674 participants with depressive symptoms at baseline (wave 2015), 262 participants with missing data on depressive symptoms during follow-up (wave 2018), and 679 participants who were lost to follow-up. Finally, 2,696 participants were available for analysis (Fig. 1**)**.

The inclusion criteria were: 1). age ≥ 60 years old; 2). had data on depressive symptoms and living arrangement. The exclusion criteria were: 1). had depressive symptoms at baseline in the longitudinal analysis; 2). had missing on age, sex, educational attainment, marital status, residential areas, smoking, drinking, body mass index (BMI), social activities, and financial support.

### Anthropometry and lifestyle measurements

Participants’ weights and heights were measured using standard equipment and procedures by well-trained operators. BMI was calculated as weight in kilograms divided by the square of height in meters. Demographic information and lifestyle behaviors were collected using a standard questionnaire face to face [37]. Lower education was defined as having 6 years of education or less (primary school education or illiteracy). Ever married was defined as those who had been married, divorced, widowed, or separated. Current smokers were defined as smoking at least 1 cigarette per day with the question “ever chewed tobacco, smoked a pipe, smoked self-rolled cigarettes, or smoked cigarettes/cigars” and currently smoking with the question “still has the habit or has quit” [38]. Alcohol consumption was classified as current drinkers (drank any alcoholic beverages more than once a month in the past years), and non-drinkers (used to drink any alcoholic beverages more than once a month in the past or never drink). Social activities referred to the activities that participants had done in the last month at baseline, including interacting with friends, doing voluntary or charity work, and so on. Financial support was the sum of family assistance and governmental assistance in one year before the baseline survey (in Int$). Family assistance referred to economic assistance including cash and in-kind transfers from respondents’ parents, children, siblings, and friends. Governmental assistance mainly included the public transfer income such as government subsidies, and donations or compensation. In the subgroup analysis, financial support was grouped into tertiles and defined as low, medium, and high groups.

### Definition of living alone and depressive symptoms

Living alone was defined as participants who did not live with their spouses, parents or parents-in-law, children, siblings, and spouses’ siblings ever or less than 11 months in the past year at baseline [37]. Depressive symptoms were measured using the 10-item Center for Epidemiological Studies-Depression Scale (CES-D10). The Cronbach’s α coefficient of the CES-D10 scale in the Chinese population is 0.815, which is a reliable self-rated depressive symptom measurement scale [39]. The CES-D10 scale is scored from 0 to 30 with 10 items using a four-point Likert scale [0 = rarely or none of the time (< 1 day); 1 = some or a little of the time (1–2 days); 2 = occasionally or a moderate amount of the time (3–4 days); 3 = most or all of the time (5–7 days)] [40]. In this scale, the items “I felt hopeful about the future” and “I was happy” were scored in reverse. Participants were considered as having depressive symptoms if the CES-D10 score was 10 or higher [41]. A higher score indicates severe depressive symptoms.

### Statistics methods

Continuous variables were winsorized, where values below the 2.5th percentile and values above the 97.5th percentile were set to the 2.5th and 97.5th percentiles, respectively [42]. Continuous variables were expressed as means ± standard deviation (x̄ ± *s*), and categorical variables were reported as percentages (*n* [%]). The two-sample *t*-test and the χ^2^ test were used to analyze continuous variables and categorical variables, respectively. Participants’ financial support was not a normal distribution and was expressed as *M(P*_*25*_, *P*_*75*_*)*, and the *Wilcoxon* rank-sum was applied to analyze the difference between participants living alone and living with others.

Multivariate Logistic regression was used to estimate the odds ratios (ORs) and 95% confidence intervals (CIs) between living alone and depressive symptoms. Model 1 was a univariate analysis; Model 2 was adjusted for age and sex; Model 3 was further adjusted for BMI, residential areas, education levels, smoking, drinking, social activities, and financial support. Model 4 was additionally adjusted for marital status.

Stratified analyses were performed by the following factors: sex (male vs. female), age (< 70 vs. ≥70 years old), BMI (< 24 vs. ≥24 kg/m^2^), residential areas (rural vs. urban), educational attainment (< 6 vs. ≥6 years), current smoking (Yes vs. No), current alcohol consumption (Yes vs. No), social activity(Yes vs. No), and financial support (low, medium, and high). Potential interactions were assessed by adding interaction terms between the above grouping factors and living alone to the Logistic models.

In a sensitivity analysis, CES-D10 scores were treated as a continuous variable, and linear regression was adopted. Moreover, multiple imputation was used to evaluate the impact of missing data. All analyses were performed with STATA SE 16.0 (StataCorp., TX, US). A two-sided *P* < .05 was considered statistically significant.

## Results

### General characteristics

In the cross-sectional analysis, 5,311 participants without missing data were included, who were aged 68.7 ± 6.8 years and 2,353(44.3%) were females. Of them, 963(18.1%) participants lived alone and 1,921(36.2%) participants were defined as having depressive symptoms. After excluding the participants with depressive symptoms at baseline and those participants with missing data, a total of 2,696 participants (mean age was 68.0 ± 6.3 years and 37.8% were females) were included in the cohort analysis, of them, 387(14.4%) participants were categorized as living alone at baseline and 848(31.1%) were identified as having depressive symptoms after a three-year follow-up. There were statistically significant differences in age, sex, residential areas, marital status, education levels, current drinking, and financial support between participants who lived alone and participants who lived with others in both cross-sectional and cohort analysis (*P* < .05). BMI was only statistically significant in cross-sectional analysis (Table 1).

**Table 1:** Characteristics of participants (n_1_ = 5 311 for the cross-sectional analysis, and n_2_ = 2 696 for the cohort analysis)

Subjects	Cross-sectional analysis (n = 5 311)			Cohort analysis (n = 2 696)		
	Living with others (n = 4 348)	Living alone (n = 963)	*P* value	Living with others (n = 2 309)	Living alone (n = 387)	*P* value
Age (years)	68.2 ± 6.5	71.3 ± 7.6	< 0.001^**^	67.6 ± 6.0	70.3 ± 7.2	< 0.001^**^
Females (%)	1,808 (41.6)	545(56.6)	< 0.001^**^	825(35.7)	195(50.4)	< 0.001^**^
Body mass index (kg/m^2^)	23.4 ± 3.6	23.1 ± 3.5	0.004^*^	23.6 ± 3.5	23.3 ± 3.3	0.131
Living in rural areas (%)	3,298(75.9)	794(82.5)	< 0.001^**^	1,694(73.4)	303(78.3)	< 0.001^**^
Education (≤ 6 years, %)	2,158(49.6)	623(64.7)	< 0.001^**^	991(42.9)	218(56.3)	< 0.001^**^
Married (%)	4,327(99.5)	915(95.0)	< 0.001^**^	2,301(99.7)	375(96.9)	< 0.001^**^
Current smokers (%)			0.688			0.539
No	3,019(69.4)	675(70.1)		1,558(67.5)	255(65.9)	
Yes	1,329(30.6)	288(29.9)		751(32.5)	132(34.1)	
Current drinkers (%)			< 0.001^**^			0.008^*^
No	2,773 (63.8)	676(70.2)		1,374(59.5)	258(66.7)	
Yes	1,575(36.2)	287(29.8)		935(40.5)	129(33.3)	
Social activities (%)	2,257(51.9)	503(52.2)	0.856	1,262(54.7)	206(53.2)	0.602
Financial support (Int$)	917.3(297.2,2 930.0)	775.2(258.4,1 576.2)	< 0.001^**^	1 033.6(335.9,2 325.6)	801.0(271.3,1 633.1)	< 0.001^**^
Depressive symptoms (%)	1 496(34.4)	425(44.1)	< 0.001^**^	686(29.7)	152(39.3)	< 0.001^**^
CES-D10 (points)	8.3 ± 6.4	9.8 ± 7.1	< 0.001^**^	7.4 ± 6.1	9.0 ± 7.1	< 0.001^**^

CES-D10, 10-item Center for Epidemiological Studies-Depression Scale

^*^P < 0.05; ^**^P < 0.001

### Association between living alone and depressive symptoms

**Fig. 2:**
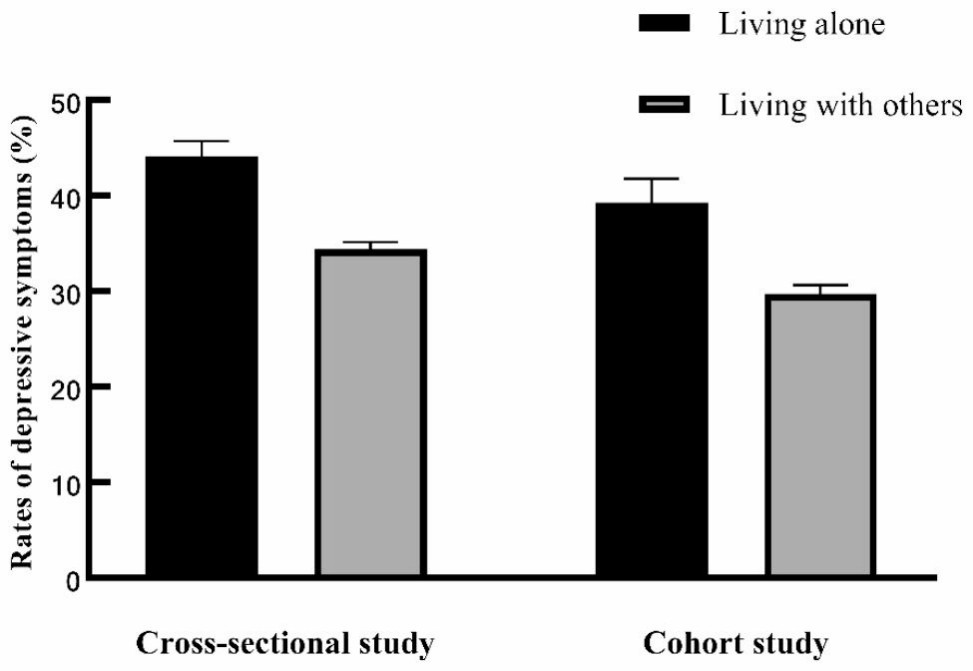
Prevalence and incidence of depressive symptoms in people living alone and living with others. The error bars represent the standard error of measurements (n_1_ = 5 311 for the cross-sectional study, and n_2_ = 2 696 for the cohort study)

The prevalence and incidence of depressive symptoms in participants who lived alone were significantly higher than those who lived with others in both cross-sectional (*P* < .001) and cohort analysis (*P* < .001) (Fig. 2). After adjusting for age, sex, education levels, smoking, drinking, BMI, residential areas, social activities, and financial support participants who lived alone had a higher risk of depressive symptoms (OR: 1.33; 95%CI: 1.14,1.54; *P* < .001 for cross-sectional analysis; OR: 1.23; 95%CI: 0.97,1.55; *P* = .084 for cohort analysis). The result remained similar even after adjusting for marital status (OR: 1.30; 95%CI: 1.12,1.52; *P* < .001 for cross-sectional analysis; OR: 1.24; 95%CI: 0.98,1.57; *P* = .069 for cohort analysis) (Table 2).

**Table 2:** Association between living-alone and depressive symptoms (n_1_ = 5 311 for the cross-sectional analysis, and n_2_ = 2 696 for the cohort analysis)

	Cross-sectional analysis		Cohort analysis	
	OR (95% CI)	*P* value	OR (95% CI)	*P* value
Model 1	1.51 (1.31,1.40)	< 0.001^**^	1.53 (1.22,1.91)	< 0.001^**^
Model 2	1.43 (1.23,1.65)	< 0.001^**^	1.36 (1.08,1.71)	0.009^*^
Model 3	1.33 (1.14,1.54)	< 0.001^**^	1.23 (0.97,1.55)	0.084
Model 4	1.30 (1.12,1.52)	0.001^*^	1.24 (0.98,1.57)	0.069

Model 1: Unadjusted

Model 2: Adjusted for Model 1 + age + sex

Model 3: Adjusted for Model 2 + education levels, smoking, drinking, body mass index, residential areas, social activities and financial support

Model 4: Adjusted for Model 3 + marital status

OR, odds ratio; CI, confidence interval

^*^P < 0.05; ^**^P < 0.001

### Subgroup analysis for cohort analysis

**Fig. 3:**
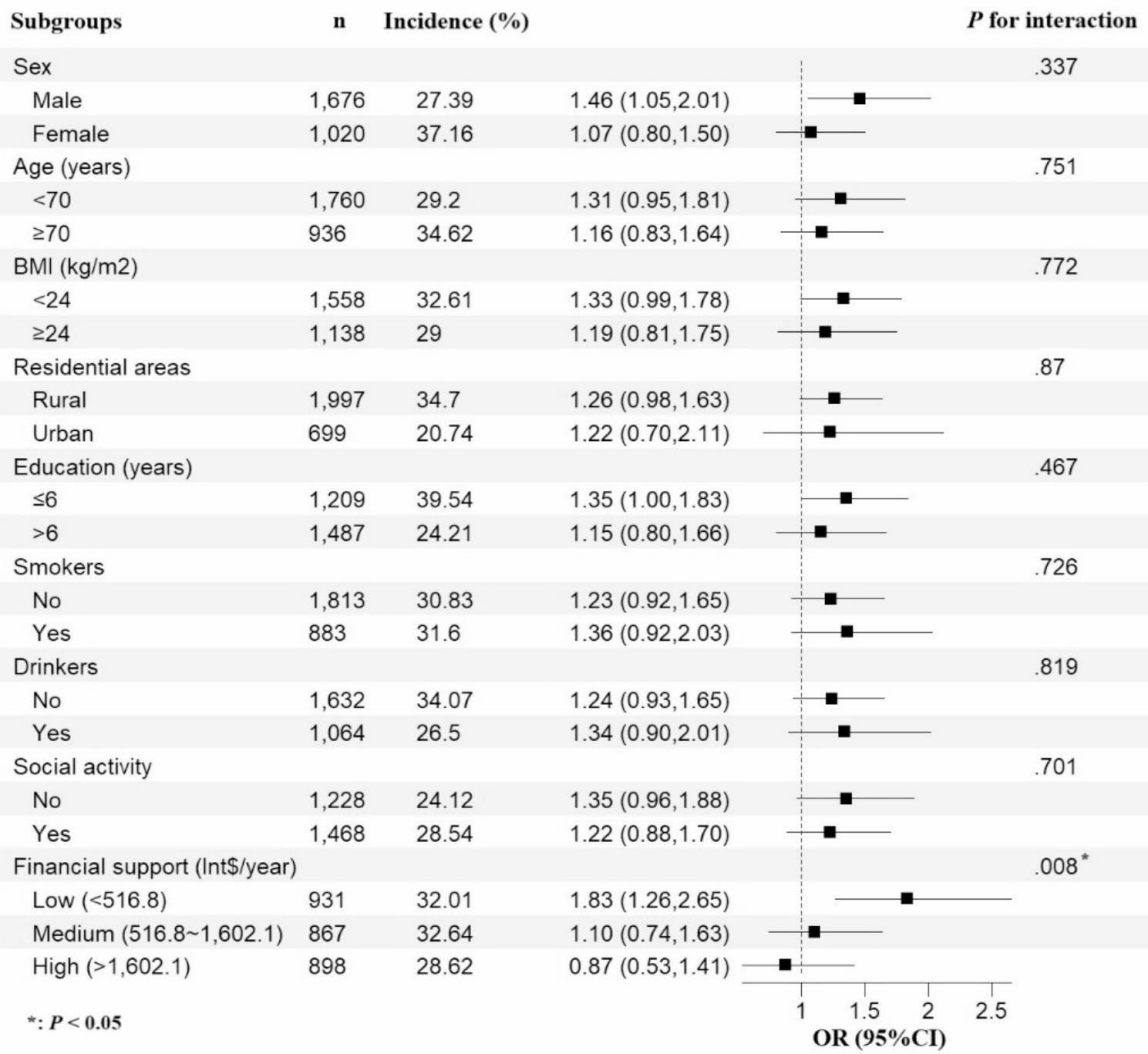
Result of subgroup analysis for cohort data. The small squares represent the odds ratio values, and the error bars represent the 95% confidence intervals for odds ratio in each subgroup (n = 2 696)

There was a statistically significant interaction between financial support and living alone (*P*_interaction_ = 0.008) on the risk of depressive symptoms. To be specific, after adjusting for other covariates, compared to those who lived with others, the risk of depressive symptoms in participants who lived alone increased by 83% (OR: 1.83; 95%CI: 1.26,2.65; *P* = 0.001) in participants with low financial support. However, we did not find statistically significant associations in participants with medium (OR: 1.10; 95%CI: 0.74,1.63; *P* = 0.647) and higher financial support (OR: 0.87; 95%CI: 0.53,1.41; *P* = 0.561) **(**Fig. 3**)**. We additionally analyzed the influence of family support and governmental support, respectively, and the results showed a similar trend. Further, financial support from families appeared to have a greater influence on the association between living alone and depressive symptoms in the elderly (see Supplementary Table 1 in the supplementary file**).** Stratified analyses by the following factors: age, sex, BMI, residential area, education level, social activity, smoking, and drinking status showed largely parallel trends.

### Sensitivity analysis

We further investigated the association between living alone and CES-D10 scores and the result was consistent (see Supplementary Table 2 in the supplementary file). Additionally, the association between living alone and depressive symptoms did not change significantly by using multiple imputation data (see Supplementary Table 3 in the supplementary file).

## Discussion

In this study, we investigated the association between living alone and depressive symptoms using cross-sectional and longitudinal data from a representative Chinese population. The result showed that living alone was significantly associated with a higher risk of depressive symptoms. Further, a significant interaction between financial support and living alone on the risk of depressive symptoms was observed. In the stratified analysis, the risk of depressive symptoms in participants who lived alone increased by 83% in participants receiving low financial support. However, we did not find statistically significant associations in participants receiving medium and higher financial support.

Our results were consistent with most of the prior cross-sectional studies. For example, a meta-analysis included 16 cross-sectional studies showed that the elderly living alone had a 1.44 times higher hazard for later life depression than those who had other living arrangements [43]. Additionally, a cohort study found that living alone significantly increased the risk of depression among Koreans aged 45 years and older [44]. Another cohort study from Japan indicated that living alone not only affected participants’ depressive symptoms but also their sense of well-being [45]. Its result also suggested that the risk of depressive symptoms was lower in people living in urban areas [45], which was in line with our findings to some extent. While Kim et al. reported that respondents residing in rural areas had a lower risk of having depressive symptoms [24]. A possible explanation is the differences of harmonious interpersonal relationships and social support between people living in rural and urban areas among different countries [46, 47].

We, for the first time, reported that financial support may help to mitigate the negative effects of living alone on depressive symptoms. The stress theory may explain this phenomenon to some extent. It hypothesizes that the influence of stress on depression can be buffered by personal resources [48]. Old adults with low financial support levels are more likely to be in disadvantaged socioeconomic conditions, so they have inadequate resources to relieve this stress [49]. In addition, we further found that family financial support may have a greater influence on minimizing the risk of depressive symptoms from living alone than government support in the elderly, which was consistent with another study [50]. Low frequency of contact with families has been proven to be a risk factor for depression in old adults [51]. In China, more financial support (including cash and in-kind) is associated with higher visiting frequency from elderly people’s children, families, and friends. Emotional communication during visiting serves as a buffer for stressful environments indirectly and protects from depression [52]. In this process, the elderly who lived alone received not only financial support but emotional assistance. The findings suggested that more attention should be paid to the living-alone situation in people with low levels of financial support.

The underlying mechanisms of how living alone influence depressive symptoms are still not well understood. Studies indicated that loneliness played an indispensable role in this process [53–55]. Living alone may lead to smaller social networks, less social support, and more social isolation, and they have a stronger sense of loss when compared with those living with others [19]. This results in a greater risk of depressive symptoms. On the other hand, participants with higher levels of loneliness were more likely to have disturbances in the structural brain networking, including the superior frontal gyrus and amygdala [56], which could increase the risk of depressive symptoms [57]. Moreover, perceived loneliness and isolation could cause impaired sleep, increased systemic inflammation, and increased sympathetic tone and cause worse depressive symptoms in return [10, 55].

This study was among the first to investigate the longitudinal association between living alone and depressive symptoms in the general Chinese population aged 60 and above with a large sample size. We, for the first time, found that higher financial support levels may attenuate the effect of living alone on the risk of depressive symptoms. Our study also has several limitations. First, living alone is highly related to loneliness, but we have no way to clearly distinguish their effect because the question about loneliness was included in the CES-D10 scale. Second, participants’ living arrangements were not collected in wave 2018, so we could not know whether their living arrangements changed or not during the follow-up period. In addition, the observational nature of this study is not sufficient to infer causality. Besides, we cannot fully exclude the possibility that the observed association is due to residual confoundings, such as the duration of living alone, frequency of meeting with family, and socioeconomic factors [51]. Finally, further studies must assess the generalizability of our findings across diverse contexts, cultures, and populations.

## Conclusion

In conclusion, our result demonstrated that living alone was associated with a higher risk of depressive symptoms, especially in a population receiving lower financial support. As an effective and easy predictor, living alone may be useful for the early identification of high-risk populations of depression and for creating a successful risk management plan in the older population. Furthermore, the findings added more evidence that more attention should be paid to the aged population who live alone.

### Electronic supplementary material

Below is the link to the electronic supplementary material.

Supplementary Material 1: Supplementary tables related to this article.

## Data Availability

The datasets supporting the conclusions of this article are available in the website of China Health and Retirement Longitudinal Study (https://charls.pku.edu.cn/).

## List of abbreviations

BMI: body mass index
CES-D10: 10-item Center for Epidemiological Studies-Depression Scale
CHARLS: China Health and Retirement Longitudinal Study
CIs: confidence intervals
ORs: odds ratios
