# Supplementary material for: Living alone and the risk of depressive symptoms: a cross-sectional and cohort analysis based on the China Health and Retirement Longitudinal Study
Source: BMC Psychiatry. 2023 Nov 17;23:853. doi: 10.1186/s12888-023-05370-y (PMC10655346; doi:10.1186/s12888-023-05370-y)
Supplement: Supplementary file 1 — Supplementary Material 1: Supplementary tables related to this article. [file 12888_2023_5370_MOESM1_ESM.docx]

| **Supplementary Table 1** The association between living alone and the risk of depressive symptoms by family support and government support (n = 2 696) | | | | | |
| --- | --- | --- | --- | --- | --- |
| Family support | | | Government support | | |
| Groups | OR (95% CI) | *P* value | Groups | OR (95% CI) | *P* value |
| Tertile 1 (<310.1) | 1.67 (1.12,2.51) | .013^*^ | Tertile 1 (0) | 1.32 (0.97,1.81) | .079 |
| Tertile 2 (310.1~1,292.0) | 1.17 (0.80,1.73) | .422 | Tertile 2 (0~64.6) | 1.19 (0.57,2.47) | .639 |
| Tertile 3 (>1,292.0) | 0.93 (0.59,1.48) | .768 | Tertile 3 (>64.6) | 1.12 (0.73,1.71) | .606 |
| Adjusted for age, sex, education levels, smoking, drinking, body mass index, residential areas, and social activities.  ^*^: *P*<.05; ^**^: *P*<.001 | | | | | |

| **Supplementary Table 2** Association between living alone and CES-D10 scores (treated as continuous variable) | | | | |
| --- | --- | --- | --- | --- |
|  | Cross-sectional study (n =5 311) | | Cohort study (n = 2 696) | |
|  | β (95% CI) | *P* value | β (95% CI) | *P* value |
| Model 1 | 1.53 (1.07,1.98) | <.001^**^ | 1.65 (0.98,2.32) | <.001^**^ |
| Model 2 | 1.27 (0.81,1.73) | <.001^**^ | 1.23 (0.56,1.91) | <.001^**^ |
| Model 3 | 0.92 (0.47,1.38) | <.001^**^ | 0.89 (0.22,1.56) | .009^*^ |
| Model 4 | 0.86 (0.40,1.32) | <.001^**^ | 0.90 (0.23,1.57) | .008^*^ |
| Model 1: Unadjusted;  Model 2: Adjusted for Model 1 + age + sex;  Model 3: Adjusted for Model 2 + education levels, smoking, drinking, body mass index, residential areas, social activities and financial support; Model 4: Adjusted for Model 3 + marital status;  OR=odds ratio; CI=confidence interval  ^*^: *P*<.05; ^**^: *P*<.001 | | | | |

| **Supplementary Table 3** Association between living-alone and depressive symptoms (using imputed data) | | | | |
| --- | --- | --- | --- | --- |
|  | Cross-sectional study (n = 5 396) | | Cohort study (n = 2 958) | |
|  | OR (95% CI) | *P* value | OR (95% CI) | *P* value |
| Model 1 | 1.50 (1.30,1.72) | <.001^**^ | 1.48 (1.20,1.82) | <.001^**^ |
| Model 2 | 1.42 (1.22,1.64) | <.001^**^ | 1.27 (1.02,1.57) | .029^*^ |
| Model 3 | 1.31 (1.13,1.52) | <.001^**^ | 1.15 (0.92,1.43) | .216 |
| Model 4 | 1.29 (1.11,1.50) | .001^*^ | 1.17 (0.93,1.46) | .173 |
| Model 1: Unadjusted;  Model 2: Adjusted for Model 1 + age + sex;  Model 3: Adjusted for Model 2 + education levels, smoking, drinking, body mass index, residential areas, social activities and financial support; Model 4: Adjusted for Model 3 + marital status;  OR=odds ratio; CI=confidence interval  ^*^: *P*<.05; ^**^: *P*<.001 | | | | |

| **Supplementary Table 4** Comparison of excluded and included participants (cross-sectional study) | | | | | |
| --- | --- | --- | --- | --- | --- |
| Subjects | Overall  (n=23 937) | Excluded  (n=18 626) | Included  (n=5 311) | *P* value | |
| Age (years) | 68.74±6.82 | - | 68.74±6.82 | - |  |
| Body mass index (kg/m^2^) | 23.81±3.58 | 23.96±3.57 | 23.36±3.58 | <.001^**^ |  |
| Females (%) | 11 137 (52.38) | 8 784 (55.07) | 2 353 (44.30) | <.001^**^ | |
| Married (%) | 21 068 (99.13) | 15 826 (99.27) | 5 242 (98.70) | <.001^**^ | |
| Education (≤6 years, %) | 95 65 (44.32) | 6 784 (41.69) | 2 781 (52.36) | <.001^**^ | |
| Living in rural areas (%) | 17 269 (72.30) | 13 247 (71.32) | 4 022 (75.73) | <.001^**^ | |
| ^*^: *P*<.05; ^**^: *P*<.001 | | | | | |

| **Supplementary Table 5** Comparison of excluded and included participants (cohort study) | | | | |
| --- | --- | --- | --- | --- |
| Subjects | Overall  (n=5 311) | Excluded  (n=2 615) | Included  (n=3 696) | *P* value |
| Age (years) | 68.74±6.82 | 69.55±7.23 | 67.95±6.29 | <.001^**^ |
| Body mass index (kg/m2) | 23.36±3.58 | 23.16±3.64 | 23.55±3.52 | <.001^**^ |
| Females (%) | 2 353 (44.30) | 1 333 (50.98) | 1 020 (37.83) | <.001^**^ |
| Married (%) | 5 242 (98.70) | 2 566 (98.13) | 2 676 (99.26) | <.001^**^ |
| Education (≤6 years, %) | 2 781 (52.36) | 1 572 (60.11) | 1 209 (44.84) | <.001^**^ |
| Living in rural areas (%) | 4 092 (77.05) | 2 095 (80.11) | 1 997 (74.07) | <.001^**^ |
| ^*^: *P*<.05; ^**^: *P*<.001 | | | | |
